# Methylation array data can simultaneously identify individuals and convey protected health information: an unrecognized ethical concern

**DOI:** 10.1186/1868-7083-6-28

**Published:** 2014-11-19

**Authors:** Robert A Philibert, Nicolas Terry, Cheryl Erwin, Winter J Philibert, Steven RH Beach, Gene H Brody

**Affiliations:** Department of Psychiatry, University of Iowa, Rm 2-126 MEB, 500 Newton Road, Iowa City, IA 52242 USA; Behavioral Diagnostics Inc, 316 E. Court St, Iowa City, IA 52244 USA; Indiana University, Robert H. McKinney School of Law, 530 W. New York St, Indianapolis, IN 46202 USA; Departments of Medical Education and Psychiatry, Texas Tech University Health Sciences Center, 3601 4th St, Lubbock, TX 79430 USA; Center for Family Research, University of Georgia, 1905 College Station Road, Athens, GA 30602 USA; Department of Psychology, University of Georgia, 125 Baldwin St, Athens, GA 30602 USA

**Keywords:** Genetics, DNA methylation, Ethics, Methylation array, Confidentiality

## Abstract

**Background:**

Genome-wide methylation arrays are increasingly used tools in studies of complex medical disorders. Because of their expense and potential utility to the scientific community, current federal policy dictates that data from these arrays, like those from genome-wide genotyping arrays, be deposited in publicly available databases. Unlike the genotyping information, access to the expression data is not restricted. An underlying supposition in the current nonrestricted access to methylation data is the belief that protected health and personal identifying information cannot be simultaneously extracted from these arrays.

**Results:**

In this communication, we analyze methylation data from the Illumina HumanMethylation450 array and show that genotype at 1,069 highly informative loci, and both alcohol and smoking consumption information, can be derived from the array data.

**Conclusions:**

We conclude that both potentially personally identifying information and substance-use histories can be simultaneously derived from methylation array data. Because access to genetic information about a database subject or one of their relatives is critical to the de-identification process, this risk of de-identification is limited at the current time. We propose that access to genome-wide methylation data be restricted to institutionally approved investigators who accede to data use agreements prohibiting re-identification.

**Electronic supplementary material:**

The online version of this article (doi:10.1186/1868-7083-6-28) contains supplementary material, which is available to authorized users.

## Background

The balance between the right to privacy and the public interest in advancing medical science is a dynamic relationship. This is particularly true for studies of complex medical disorders. Over the past decade, vast databases of biological information, both private and governmental, have been established. A major factor in their rapid growth has been policies mandating the deposition of all genome-wide array data in publicly available repositories, such as those administered by the National Center for Biotechnology Information (NCBI) [1].

Without a doubt, these policies have led to significant advances in many areas including evolutionary biology and healthcare. With respect to medical illness, repositories of genome-wide genetic data are routinely utilized in meta-analyses of cardiopulmonary, endocrinological and mental health disorders. An underlying supposition in making genome-wide genetic data publicly available is the belief that the information contained within them cannot be used to both infer disease status and uniquely identify individuals. The rationale for the first assumption rests on a firm foundation of medical evidence which shows that for the vast majority of non-autosomal dominant disorders, genetic information alone cannot be used to absolutely determine whether a given individual actually has a medical disorder. For the more common complex disorders, this is undoubtedly true. For example, with respect to Type 2 Diabetes (T2DM), which is perhaps the best understood common complex medical disorder from a genetic point of view, individual genotyping data is of relatively little value in determining whether a given individual is at risk, let alone currently ill [2, 3]. With respect to the latter supposition, there was an erroneous expectation that anonymized genome-wide genetic data contained within repositories could not be linked to identifiable individuals.

Recent developments in bioinformatics have shown clearly that the assumption that genotype data in public repositories cannot be tied to identifiable individuals is not correct in all cases. For example, Gymek and colleagues reported a method to triangulate the identity of a sample donor using genomic data and surnames from publicly available databases [4]. This development, in combination with other isolated but related issues, such as the sequencing of the commonly used HeLa cell line, which unintentionally allowed the conveyance of the likely genetic vulnerabilities of close relatives of Henrietta Lacks, have led to changes in the way genome-wide genetic information is handled [5]. However, the severity of these concerns has been tempered by the fact that, with the exception of isolated instances, protected information regarding diseases status has not been compromised. That is, the ability of genome-wide data to both uniquely identify an individual and infer disease status is relatively limited. Nevertheless, as a precaution, access to full genetic information is restricted [6].

In contrast, the deposition of genome-wide methylation data, such as that of the Illumina HumanGenome450 BeadArray to the Gene Omnibus Expression (GEO) repository has largely escaped scrutiny [7]. The rationale for this relative lack of concern is the unwritten supposition that methylation data cannot be used to uniquely identify individuals or convey sensitive protected health information.

Unfortunately, emerging data indicate that both of these assumptions may be incorrect. Recently, our consortium has demonstrated that consumption histories of both tobacco and alcohol can be accurately inferred from the DNA methylation signature of peripheral white blood cells [8–11]. In this communication, we describe a method by which information from a DNA methylation array could be used to generate individually identifying genetic profiles and also to infer the substance-use consumption of study participants. We then discuss the potential for the misuse of this data by those with access to genetic information of the study participants or their close relatives.

## Results

The suitability of Illumina array DNA methylation data for use in genotyping was explored in two ways. As a first approximation of the total variation, the beta values for all 21,969 probes mapping to chromosome 16 were plotted and visually inspected for possible genetic influences on methylation. Overall, 707 probes displayed a tri-modal distribution roughly consistent with an additive effect of genotype on DNA methylation.

In the more exacting second approach to inferring genotype, we attempted to build on the prior observations by Shoemaker and colleagues who noted nearly complete, stoichiometric losses of the methylation signal in response to cytosine polymorphisms in heavily methylated (approximately 95 to 100%) CpG residues, [12] by identifying those sites with ideal beta value distributions for genotyping inference (that is, 100%, 50% or 0% methylation). First, the cleaned beta values for all 485,577 loci interrogated by the Illumina HumanMethylation450 array in our recent study of the effects of smoking on DNA methylation in a cohort of 111 African American females were binned into three groups, X, Y and Z (X >0.7, 0.7 < Y <0.3, Z <0.25), which potentially represent the genotypes CC, CD, or DD where ‘D’ represents an A, G or T, at the putative cytosine position of the CpG dinucleotide pair, respectfully [13]. A total of 1,383 probes with at least 106 (95%) of the values mapping to those bins and having at least four observations in both Bin X and Bin Z were identified. The binning values were converted to genotypes and then analyzed for minor allele frequency and compliance with the Hardy Weinberg Equilibrium (HWE). A total of 1,069 had HWE *P* values >0.01, with their minor allele frequencies ranging from 0.105 to 0.5. A listing of the 30 most informative loci is given in Table 1, and a complete listing of all 1,069 probes, including probe sequence information is given in Additional file 1: Table S1. Histograms of the beta value distribution at 30 most informative loci are given in Additional file 2: Figure S1.

**Table 1:** **Location and heterozygosity of the top thirty Illumina probes**

Illumina ID	CHR	Position (bp) ^1^	Observed heterozygosity	UCSC ^2^heterozygosity
cg11036359	6	29759078	0.55	0.45
cg03115532	6	28185726	0.46	0.43
cg10695549	8	18432000	0.53	0.50
cg22309983	17	3497580	0.53	0.31
cg09533869	8	97747124	0.48	0.45
cg13078798	1	92203667	0.41	0.46
cg23603995	6	157198648	0.54	0.41
cg27467876	8	22266134	0.50	0.50
cg27625131	13	113105794	0.58	0.21
cg26690318	10	100167465	0.56	0.46
cg16999994	11	1001560	0.56	0.50
cg27056740	14	101507727	0.56	0.49
cg18816122	5	164064	0.45	0.50
cg13821051	2	101124858	0.52	0.46
cg06688803	19	45457306	0.46	0.49
cg18662228	2	236867804	0.52	0.50
cg27076160	10	64431533	0.62	0.30
cg22953237	7	31425682	0.44	0.02
cg16814680	8	91681699	0.44	0.50
cg18239511	14	96563269	0.55	0.39
cg13379757	10	22717154	0.46	0.30
cg19214707	7	3157722	0.48	0.19
cg11019791	22	48896579	0.48	0.45
cg04506342	2	160463692	0.51	0.48
cg25046571	6	29794657	0.51	0.50
cg10117599	7	624424	0.51	0.48
cg16398051	15	100821466	0.51	0.50
cg18514595	22	49579968	0.51	0.49
cg16675926	1	233518998	0.50	0.49
cg02299007	8	1140574	0.46	0.48

^1^Position of CpG residue according to Genome Build 37.

^2^Heterozygosity as reported by UCSC Genome Browser.

Using the sequence information contained in the Illumina probe annotation files and the sequence alignment algorithm of the University of California, Santa Clara (UCSC) Genome Browser, we mapped the CpG residue targeted by each of the probes back to the genome to determine whether or not the position occupied by the cytosine nucleotide was known to be polymorphic. In 29 of the top 30 cases, the position of the CpG residue targeted by the probe was the site of a known highly informative C to T transition polymorphism whose USCS Genome Browser-listed heterozygosity closely matched that observed in our study. The sole exception in that group of 30 was with respect to cg19214707, which instead contained several polymorphisms within the probe binding site. A review of a random sampling of the rest 1,069 probes listed in Additional file 1: Table S1 showed a high correlation between the heterozygosity observed in our sample of 111 subjects and that reported on the UCSC genome browser.

To formally determine whether allele binning of methylation signal corresponded to actual genotypes, we genotyped 12 random Family and Community Health Studies (FACHS) subjects at two of the loci (cg10695549 and cg21028319) using conventional MspI restriction endonuclease digestion. The results from each of the agarose gel based assessments showed complete correspondence to that imputed from the arrays.

**Figure 1:**
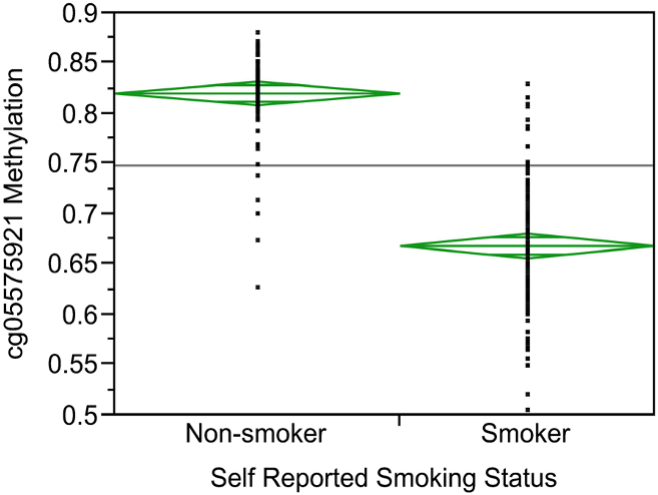
**The relationship between DNA methylation at cg05575921 and self-reported Smoking Status.**

As a demonstration of the usefulness of these arrays to assess substance-use consumption status for those unfamiliar, we repeated our previously published analysis of the relationship of methylation at cg05575921 to self-reported smoking status in these 111 individuals. We and others have shown that assessment of methylation status at cg05575921, which targets a CpG residue in the aryl hydrocarbon receptor repressor, can be used to assess smoking history [8–11, 13–18]. Figure 1 illustrates the relationship between smoking status and DNA methylation at cg05575921. As the figure demonstrates, methylation status at this residue is highly correlated with self-reported smoking status.

## Discussion

The frequent use of information from public databases in many of the most highly cited scientific papers highlights the value of these repositories. Nevertheless, recent advances in our ability to infer information about the human subject participants who contributed to those studies raises concerns for potential abuse of that information.

At the current time, the capability to link the disease status information contained within these arrays to individuals is relatively limited. However, as the current exercise demonstrates, this is not due to the lack of potential genetic markers in the Illumina array. Our rudimentary analysis that focused on identifying highly informative loci that were fully methylated, half methylated or fully demethylated as a function of genetic variation at or near the CpG site, generated over 1,000 informative loci. However, this is likely a gross underestimate of the number of informative loci. Our manual survey of chromosome 16 shows that if the stringency was relaxed to include those sites that were normally not fully methylated or were less informative than those in Table 1, the number of potential loci of that could be used to infer genotype would markedly increase. Indeed, Shoemaker noted over 200,000 annotated SNPs that map to CpG sites [12]. Hence, it is highly likely many more genetically informative probes could be identified if more advanced methods were use to ‘bin’ alleles.

Because a relatively small set of loci can be used to match one genetic sample to another, it should be possible using the thousand or so markers that we have already identified, to develop a robust, unique, genetic profile of any anonymous genome-wide methylation array donor. To use that genetic information to identify an individual person, one would need either a genetic sample of the individual to match, or else one would need to know that a given individual participated in a methylation study and have genetic information from a close relative, such as a sibling or first cousin. In our opinion, this is not a likely possibility at the current time. The GEO database only list 3,559 arrays of human peripheral blood methylation. Still, given the rapid growth of this database, on-line genetic information and the bitterness sometimes seen in divorce proceedings or other situations in which considerable sums of money or prestige are involved, it is not inconceivable that this could happen in the future. Furthermore, if our ability to identify individuals in open access genetic databases accelerates or if individuals begin to make non-anonymized genomes more common, the ability to identify those who have contributed to genome-wide methylation studies will become correspondingly easier.

Not everyone who has contributed to these databases needs to be alarmed. Although the current status of a number of medical disorders can be imputed from these arrays is growing, [19, 20] it is the recent demonstrations that these methylation arrays can be used to infer substance-use histories that may cause the greatest concerns. With respect to mental health information, only tobacco and alcohol consumption information can be accurately gleaned from these arrays at the current time [9, 21]. However, it is highly likely that our ability to assess other substance consumption, such as that of cannabis use, or other mental health status will be developed in the near future.

Currently, the identities of the donors of these data are not protected from discovery. Protection under the Healthcare Improvement and Portability Act (HIPAA) Privacy rule only applies to protected health information held by covered entities such as health care institutions [22]. However, to be protected the information must be ‘individually identifiable.’ HIPAA does not generally protect data held in publicly available repositories because the Privacy rule generally does not apply to de-identified health information [23]. The current demonstration that extensive genotype and substance-use profiles can be extracted from these arrays challenges this de-identification hypothesis and calls into question the lack of privacy protection.

The risk of re-identification is well known and should not take precedence over the rights of individual research subjects [24]. In 2010, Benitez and Malin quantified substantial differential risks of re-identification based on state-by-state variations in voter registries (their chosen triangulation datasets) [25]. The narrower question of potentially identifying genetic information has been the subject of several proposals for regulatory reform. For example, in 2009 an Institute of Medicine committee advocated ‘a focus on strong security measures and the adoption of strict prohibitions and legal sanctions against the unauthorized re-identification of individuals from DNA sequences.’ [26]. In 2012, the Presidential Commission for the Study of Bioethical Issues called for more consistent privacy baseline rules and a focus on data security [27]. Neither report resulted in regulatory reform.

There are several potential solutions to the re-identification risks posed by methylation data. One of them is to require data use agreements and to restrict access of array or similarly informative data to those investigators appropriately vetted by their institutions. There are many examples of data use prohibitions on re-identification, such as the National Practitioner Data Bank’s conditions for the use of its Public Use Data File [28]. Another would be to remove the data for the most genetically informative markers from the database. Though the two approaches are not mutually exclusive, vetting of applicants might be the preferred mechanism. Institutional Review Boards are already available to the vast majority of researchers who would seek this type of data. It is also possible to implement the second mechanism as well. However, if this mechanism is to be completely effective ethnically inclusive examinations to determine which data should be removed must be undertaken.

## Conclusions

In summary, we report that both highly informative genetic profiles and substance-use histories can be developed from the same Illumina HumanGenome450 arrays. We suggest that policy changes be initiated to address potential loss of confidentiality.

## Availability of supporting data

The methylation data used in this study are freely available via the Gene Expression Omnibus repository [GEO Accession: GSE53045]. A listing of all 1069 polymorphic sites discussed in this manuscript is contained in the Additional files.

## Methods

The DNA methylation information contained in this study was derived and deposited as part of the study plan for the National Institutes for Health (NIH)-funded study ‘The Effects of Smoking on DNA Methylation in Primary Human Lymphocytes’ (R21DA034457, [GEO Accession: GSE53045]). All procedures and protocols in that study were approved by the University of Iowa Institutional Review Board. Self-reported smoking data on the 111 female subjects was obtained using an adapted version of the Semi-Structured Assessment for the Genetics of Alcoholism, Version II [29]. Biomaterial for the methylation analyses was obtained via phlebotomy at the time of the interview.

The genome-wide methylation data consists of 111 assessments of peripheral mononuclear cell DNA of 111 African-American females using the Illumina HumanMethylation450 BeadChip (Illumina, San Diego, CA, USA), which contains 485,577 probes recognizing at least 20,216 transcripts, potential transcripts or CpG islands. The procedures and protocols used in the preparation of the DNA and cleaning of the data have been described in detail previously [13].

Binning of beta values was conducted using Excel (Microsoft, Redmond, WA, USA). Plotting of data values was accomplished using JMP Version 11 (SAS, Cary, SC, USA).

Genotyping at cg10695549 and cg21028319 was conducted using a standard restriction enzyme digest approach. In brief, we searched the key sequence information provided in the probe annotation files of the 50 most informative loci to identify those with CpG sites that could be potentially recognized by the restriction enzyme MspI (which cuts at CCGG tetramers). Primers flanking each CpG site at two of these sites, F- GCTGTAATTATACATCCAGCTATGG and R- TTTTTGTTTCCCTTCTGAGC for cg10695549; and F- TTGCAAACGATGAGAACTGAG and R- CGTTTACCAGCCCATGCTA for cg21028319; were used to amplify the locus using DNA from 12 random FACHS subjects. Aliquots of the resulting PCR products were then digested using 3 *u* of MspI under the conditions suggested by the manufacturer (New England Biolabs, Ipswich, MA, USA). The resulting products were then electrophoresed on standard 2% agarose gel and the resulting genotypes called by personnel blinded to methylation allele status.

## Electronic supplementary material

Additional file 1: Table S1: A listing of 1069 Genetically Informative Methylation Probes. (XLSX 2 MB)

Additional file 2: Figure S1: Binning histograms for the 50 genetically most informative probes. (PDF 242 KB)
