# Supplementary material for: Methylation array data can simultaneously identify individuals and convey protected health information: an unrecognized ethical concern
Source: Clin Epigenetics. 2014 Nov 19;6(1):28. doi: 10.1186/1868-7083-6-28 (PMC4391334; doi:10.1186/1868-7083-6-28)
Supplement: Supplementary file 2 — Additional file 2: Figure S1: Binning histograms for the 50 genetically most informative probes. (PDF 242 KB) [file 13148_2014_91_MOESM2_ESM.pdf]

## Distributions

### cg11036359

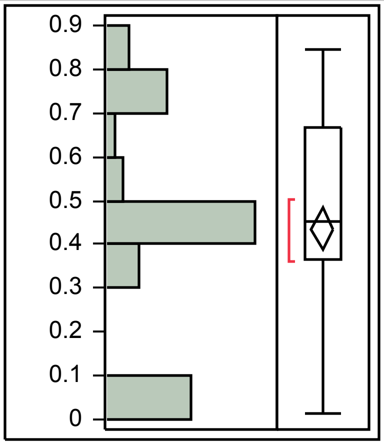


#### Quantiles

| | | |
|---|---|---|
| 100.0% | maximum | 0.84231 |
| 99.5% | | 0.84231 |
| 97.5% | | 0.82227 |
| 90.0% | | 0.78854 |
| 75.0% | quartile | 0.66615 |
| 50.0% | median | 0.45428 |
| 25.0% | quartile | 0.36757 |
| 10.0% | | 0.03138 |
| 2.5% | | 0.02511 |
| 0.5% | | 0.0131 |
| 0.0% | minimum | 0.0131 |

#### Summary Statistics

| | |
|---|---|
| Mean | 0.4363022 |
| Std Dev | 0.2589197 |
| Std Err Mean | 0.0245756 |
| Upper 95% Mean | 0.4850052 |
| Lower 95% Mean | 0.3875991 |
| N | 111 |

### cg03115532

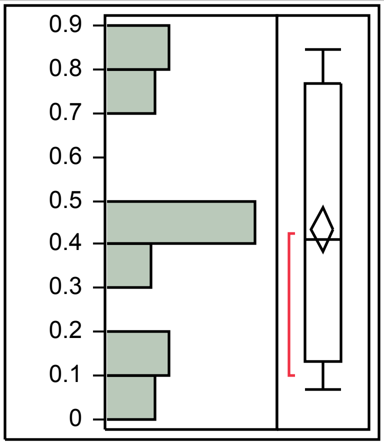


#### Quantiles

| | | |
|---|---|---|
| 100.0% | maximum | 0.84578 |
| 99.5% | | 0.84578 |
| 97.5% | | 0.83415 |
| 90.0% | | 0.81524 |
| 75.0% | quartile | 0.76902 |
| 50.0% | median | 0.41305 |
| 25.0% | quartile | 0.13178 |
| 10.0% | | 0.09232 |
| 2.5% | | 0.08177 |
| 0.5% | | 0.06832 |
| 0.0% | minimum | 0.06832 |

#### Summary Statistics

| | |
|---|---|
| Mean | 0.4341174 |
| Std Dev | 0.2609156 |
| Std Err Mean | 0.024765 |
| Upper 95% Mean | 0.4831958 |
| Lower 95% Mean | 0.385039 |
| N | 111 |

### cg10695549

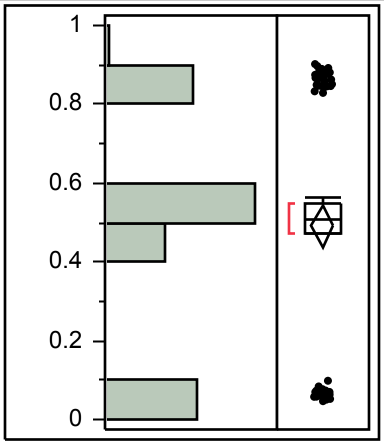


#### Quantiles

| | | |
|---|---|---|
| 100.0% | maximum | 0.90113 |
| 99.5% | | 0.90113 |
| 97.5% | | 0.89222 |
| 90.0% | | 0.87406 |
| 75.0% | quartile | 0.55022 |
| 50.0% | median | 0.50988 |
| 25.0% | quartile | 0.47276 |
| 10.0% | | 0.06448 |
| 2.5% | | 0.05262 |
| 0.5% | | 0.04757 |
| 0.0% | minimum | 0.04757 |

#### Summary Statistics

| | |
|---|---|
| Mean | 0.4905748 |
| Std Dev | 0.2763745 |
| Std Err Mean | 0.0262323 |
| Upper 95% Mean | 0.542561 |
| Lower 95% Mean | 0.4385885 |
| N | 111 |

## Distributions

### cg22309983

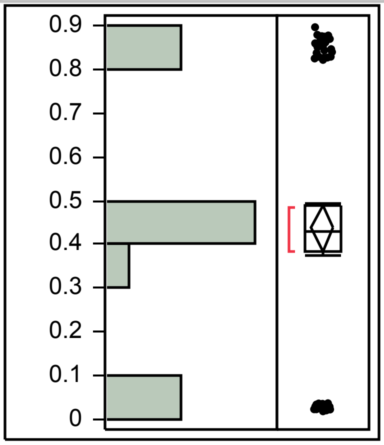


#### Quantiles

| | | |
|---|---|---|
| 100.0% | maximum | 0.89505 |
| 99.5% | | 0.89505 |
| 97.5% | | 0.87644 |
| 90.0% | | 0.85955 |
| 75.0% | quartile | 0.49044 |
| 50.0% | median | 0.4298 |
| 25.0% | quartile | 0.38207 |
| 10.0% | | 0.02857 |
| 2.5% | | 0.02314 |
| 0.5% | | 0.01982 |
| 0.0% | minimum | 0.01982 |

#### Summary Statistics

| | |
|---|---|
| Mean | 0.4364416 |
| Std Dev | 0.2841653 |
| Std Err Mean | 0.0269718 |
| Upper 95% Mean | 0.4898934 |
| Lower 95% Mean | 0.3829899 |
| N | 111 |

### cg09533869

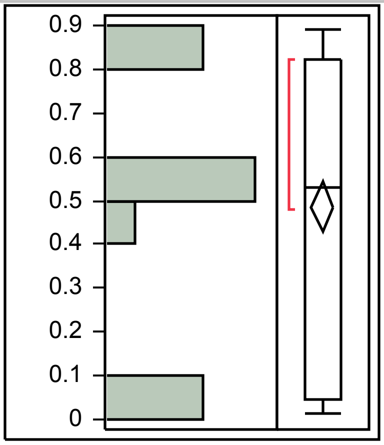


#### Quantiles

| | | |
|---|---|---|
| 100.0% | maximum | 0.89141 |
| 99.5% | | 0.89141 |
| 97.5% | | 0.88373 |
| 90.0% | | 0.87124 |
| 75.0% | quartile | 0.82117 |
| 50.0% | median | 0.52844 |
| 25.0% | quartile | 0.04625 |
| 10.0% | | 0.0258 |
| 2.5% | | 0.02051 |
| 0.5% | | 0.01643 |
| 0.0% | minimum | 0.01643 |

#### Summary Statistics

| | |
|---|---|
| Mean | 0.4854664 |
| Std Dev | 0.3045613 |
| Std Err Mean | 0.0289077 |
| Upper 95% Mean | 0.5427546 |
| Lower 95% Mean | 0.4281782 |
| N | 111 |

### cg13078798

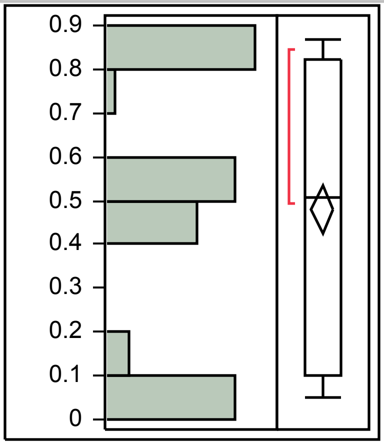


#### Quantiles

| | | |
|---|---|---|
| 100.0% | maximum | 0.86677 |
| 99.5% | | 0.86677 |
| 97.5% | | 0.86191 |
| 90.0% | | 0.84582 |
| 75.0% | quartile | 0.82004 |
| 50.0% | median | 0.50827 |
| 25.0% | quartile | 0.10032 |
| 10.0% | | 0.06618 |
| 2.5% | | 0.05293 |
| 0.5% | | 0.05033 |
| 0.0% | minimum | 0.05033 |

#### Summary Statistics

| | |
|---|---|
| Mean | 0.4801205 |
| Std Dev | 0.2934799 |
| Std Err Mean | 0.0278559 |
| Upper 95% Mean | 0.5353243 |
| Lower 95% Mean | 0.4249167 |
| N | 111 |

## Distributions

### cg23603995

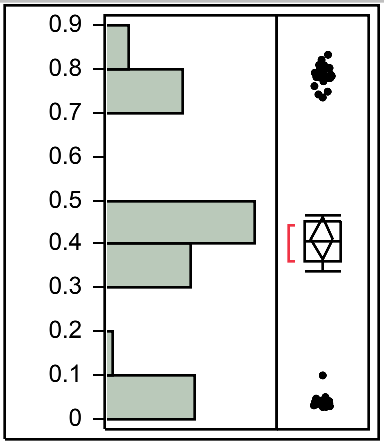


#### Quantiles

| | | |
|---|---|---|
| 100.0% | maximum | 0.83171 |
| 99.5% | | 0.83171 |
| 97.5% | | 0.81073 |
| 90.0% | | 0.78895 |
| 75.0% | quartile | 0.45115 |
| 50.0% | median | 0.40756 |
| 25.0% | quartile | 0.36155 |
| 10.0% | | 0.03735 |
| 2.5% | | 0.0296 |
| 0.5% | | 0.02901 |
| 0.0% | minimum | 0.02901 |

#### Summary Statistics

| | |
|---|---|
| Mean | 0.413532 |
| Std Dev | 0.2538835 |
| Std Err Mean | 0.0240976 |
| Upper 95% Mean | 0.4612877 |
| Lower 95% Mean | 0.3657763 |
| N | 111 |

### cg27467876

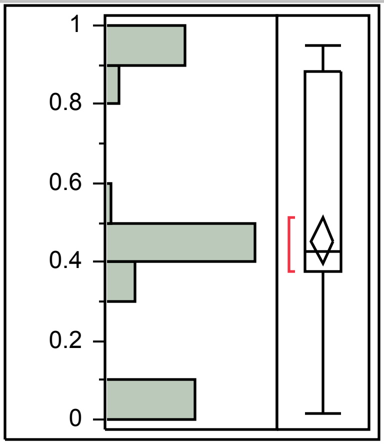


#### Quantiles

| | | |
|---|---|---|
| 100.0% | maximum | 0.94986 |
| 99.5% | | 0.94986 |
| 97.5% | | 0.93832 |
| 90.0% | | 0.91595 |
| 75.0% | quartile | 0.88009 |
| 50.0% | median | 0.42906 |
| 25.0% | quartile | 0.37651 |
| 10.0% | | 0.02762 |
| 2.5% | | 0.0215 |
| 0.5% | | 0.01801 |
| 0.0% | minimum | 0.01801 |

#### Summary Statistics

| | |
|---|---|
| Mean | 0.4541458 |
| Std Dev | 0.3143334 |
| Std Err Mean | 0.0298352 |
| Upper 95% Mean | 0.5132721 |
| Lower 95% Mean | 0.3950194 |
| N | 111 |

### cg27625131

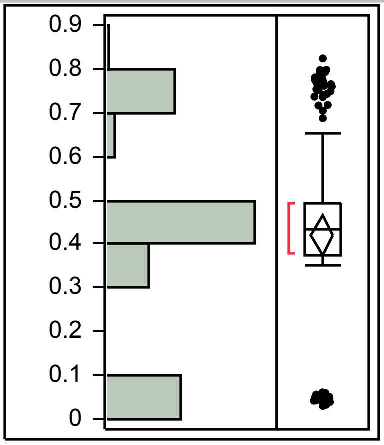


#### Quantiles

| | | |
|---|---|---|
| 100.0% | maximum | 0.82339 |
| 99.5% | | 0.82339 |
| 97.5% | | 0.79704 |
| 90.0% | | 0.76391 |
| 75.0% | quartile | 0.49495 |
| 50.0% | median | 0.43178 |
| 25.0% | quartile | 0.37658 |
| 10.0% | | 0.04858 |
| 2.5% | | 0.0387 |
| 0.5% | | 0.03161 |
| 0.0% | minimum | 0.03161 |

#### Summary Statistics

| | |
|---|---|
| Mean | 0.4212798 |
| Std Dev | 0.2383695 |
| Std Err Mean | 0.022625 |
| Upper 95% Mean | 0.4661173 |
| Lower 95% Mean | 0.3764423 |
| N | 111 |

## Distributions

### cg26690318

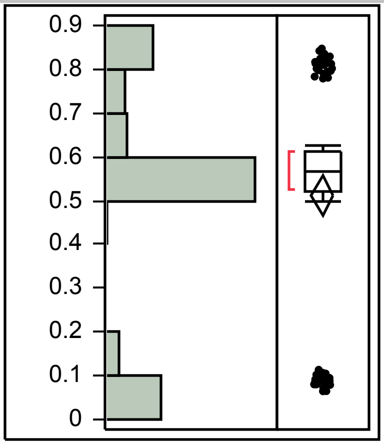


#### Quantiles

| | | |
|---|---|---|
| 100.0% | maximum | 0.84632 |
| 99.5% | | 0.84632 |
| 97.5% | | 0.83594 |
| 90.0% | | 0.81229 |
| 75.0% | quartile | 0.61104 |
| 50.0% | median | 0.56687 |
| 25.0% | quartile | 0.51993 |
| 10.0% | | 0.0878 |
| 2.5% | | 0.07199 |
| 0.5% | | 0.06549 |
| 0.0% | minimum | 0.06549 |

#### Summary Statistics

| | |
|---|---|
| Mean | 0.5111312 |
| Std Dev | 0.2491094 |
| Std Err Mean | 0.0236444 |
| Upper 95% Mean | 0.5579889 |
| Lower 95% Mean | 0.4642735 |
| N | 111 |

### cg16999994

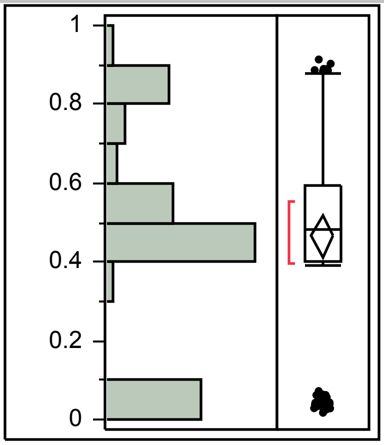


#### Quantiles

| | | |
|---|---|---|
| 100.0% | maximum | 0.91272 |
| 99.5% | | 0.91272 |
| 97.5% | | 0.89167 |
| 90.0% | | 0.87283 |
| 75.0% | quartile | 0.59365 |
| 50.0% | median | 0.48479 |
| 25.0% | quartile | 0.40303 |
| 10.0% | | 0.04079 |
| 2.5% | | 0.02778 |
| 0.5% | | 0.01824 |
| 0.0% | minimum | 0.01824 |

#### Summary Statistics

| | |
|---|---|
| Mean | 0.4649979 |
| Std Dev | 0.2724662 |
| Std Err Mean | 0.0258613 |
| Upper 95% Mean | 0.5162491 |
| Lower 95% Mean | 0.4137468 |
| N | 111 |

### cg27056740

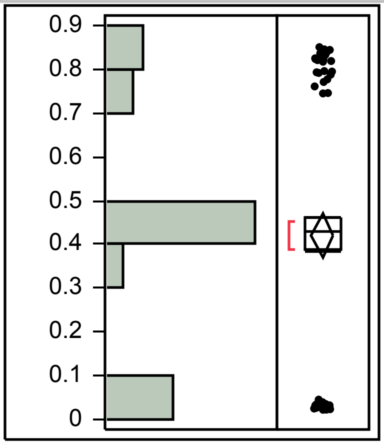


#### Quantiles

| | | |
|---|---|---|
| 100.0% | maximum | 0.84963 |
| 99.5% | | 0.84963 |
| 97.5% | | 0.84355 |
| 90.0% | | 0.82176 |
| 75.0% | quartile | 0.4606 |
| 50.0% | median | 0.43005 |
| 25.0% | quartile | 0.39055 |
| 10.0% | | 0.02986 |
| 2.5% | | 0.02426 |
| 0.5% | | 0.02325 |
| 0.0% | minimum | 0.02325 |

#### Summary Statistics

| | |
|---|---|
| Mean | 0.4199697 |
| Std Dev | 0.259707 |
| Std Err Mean | 0.0246503 |
| Upper 95% Mean | 0.4688208 |
| Lower 95% Mean | 0.3711186 |
| N | 111 |

## Distributions

### cg18816122

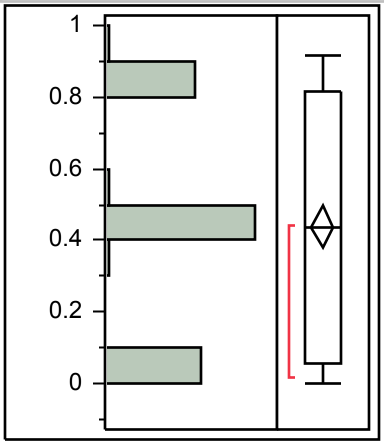


#### Quantiles

| | | |
|---|---|---|
| 100.0% | maximum | 0.91659 |
| 99.5% | | 0.91659 |
| 97.5% | | 0.88897 |
| 90.0% | | 0.86722 |
| 75.0% | quartile | 0.81625 |
| 50.0% | median | 0.43337 |
| 25.0% | quartile | 0.05659 |
| 10.0% | | 0.02119 |
| 2.5% | | 0.00955 |
| 0.5% | | 0.00215 |
| 0.0% | minimum | 0.00215 |

#### Summary Statistics

| | |
|---|---|
| Mean | 0.4378615 |
| Std Dev | 0.3084099 |
| Std Err Mean | 0.029273 |
| Upper 95% Mean | 0.4958737 |
| Lower 95% Mean | 0.3798494 |
| N | 111 |

### cg13821051

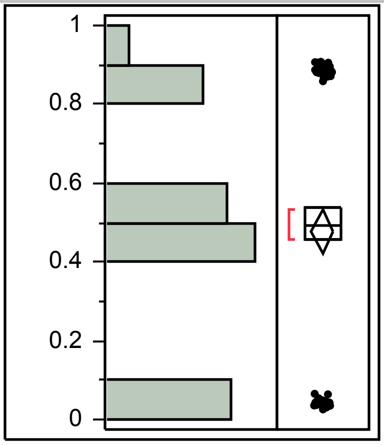


#### Quantiles

| | | |
|---|---|---|
| 100.0% | maximum | 0.90688 |
| 99.5% | | 0.90688 |
| 97.5% | | 0.90519 |
| 90.0% | | 0.88783 |
| 75.0% | quartile | 0.53699 |
| 50.0% | median | 0.49166 |
| 25.0% | quartile | 0.45602 |
| 10.0% | | 0.04206 |
| 2.5% | | 0.03263 |
| 0.5% | | 0.02673 |
| 0.0% | minimum | 0.02673 |

#### Summary Statistics

| | |
|---|---|
| Mean | 0.4779513 |
| Std Dev | 0.294087 |
| Std Err Mean | 0.0279135 |
| Upper 95% Mean | 0.5332693 |
| Lower 95% Mean | 0.4226332 |
| N | 111 |

### cg06688803

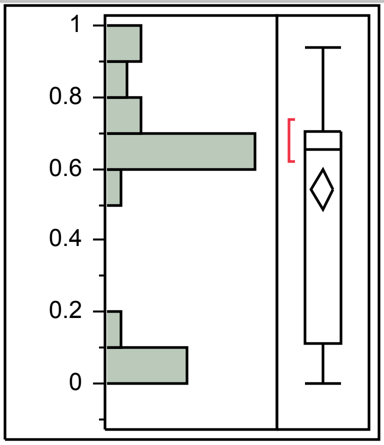


#### Quantiles

| | | |
|---|---|---|
| 100.0% | maximum | 0.93995 |
| 99.5% | | 0.93995 |
| 97.5% | | 0.92896 |
| 90.0% | | 0.90778 |
| 75.0% | quartile | 0.70178 |
| 50.0% | median | 0.65434 |
| 25.0% | quartile | 0.11387 |
| 10.0% | | 0.06245 |
| 2.5% | | 0.0223 |
| 0.5% | | 0.00387 |
| 0.0% | minimum | 0.00387 |

#### Summary Statistics

| | |
|---|---|
| Mean | 0.5413467 |
| Std Dev | 0.303886 |
| Std Err Mean | 0.0289744 |
| Upper 95% Mean | 0.598773 |
| Lower 95% Mean | 0.4839204 |
| N | 110 |

## Distributions

### cg18662228

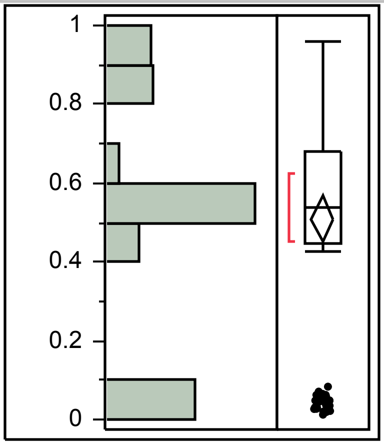


#### Quantiles

| | | |
|---|---|---|
| 100.0% | maximum | 0.95967 |
| 99.5% | | 0.95967 |
| 97.5% | | 0.93618 |
| 90.0% | | 0.91023 |
| 75.0% | quartile | 0.6792 |
| 50.0% | median | 0.53583 |
| 25.0% | quartile | 0.44618 |
| 10.0% | | 0.03623 |
| 2.5% | | 0.02091 |
| 0.5% | | 0.01335 |
| 0.0% | minimum | 0.01335 |

#### Summary Statistics

| | |
|---|---|
| Mean | 0.5098921 |
| Std Dev | 0.3011803 |
| Std Err Mean | 0.0287164 |
| Upper 95% Mean | 0.5668071 |
| Lower 95% Mean | 0.4529771 |
| N | 110 |

### cg27076160

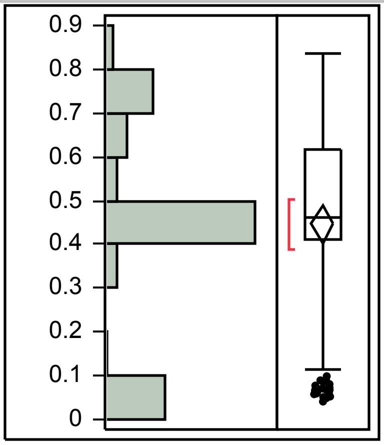


#### Quantiles

| | | |
|---|---|---|
| 100.0% | maximum | 0.83414 |
| 99.5% | | 0.83414 |
| 97.5% | | 0.81306 |
| 90.0% | | 0.75324 |
| 75.0% | quartile | 0.61505 |
| 50.0% | median | 0.46111 |
| 25.0% | quartile | 0.40969 |
| 10.0% | | 0.0685 |
| 2.5% | | 0.05155 |
| 0.5% | | 0.04192 |
| 0.0% | minimum | 0.04192 |

#### Summary Statistics

| | |
|---|---|
| Mean | 0.4461266 |
| Std Dev | 0.2240822 |
| Std Err Mean | 0.0212689 |
| Upper 95% Mean | 0.4882766 |
| Lower 95% Mean | 0.4039765 |
| N | 111 |

### cg22953237

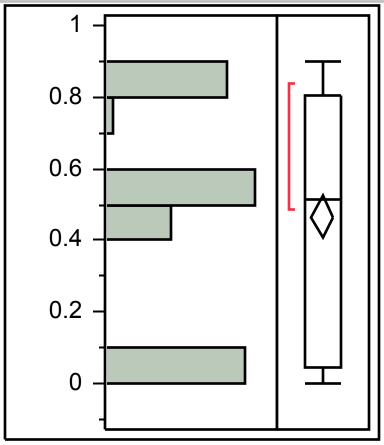


#### Quantiles

| | | |
|---|---|---|
| 100.0% | maximum | 0.89661 |
| 99.5% | | 0.89661 |
| 97.5% | | 0.87995 |
| 90.0% | | 0.85211 |
| 75.0% | quartile | 0.80249 |
| 50.0% | median | 0.51456 |
| 25.0% | quartile | 0.04738 |
| 10.0% | | 0.02854 |
| 2.5% | | 0.0154 |
| 0.5% | | 0.0034 |
| 0.0% | minimum | 0.0034 |

#### Summary Statistics

| | |
|---|---|
| Mean | 0.465507 |
| Std Dev | 0.3078581 |
| Std Err Mean | 0.0292206 |
| Upper 95% Mean | 0.5234154 |
| Lower 95% Mean | 0.4075987 |
| N | 111 |

# Distributions

## cg16814680

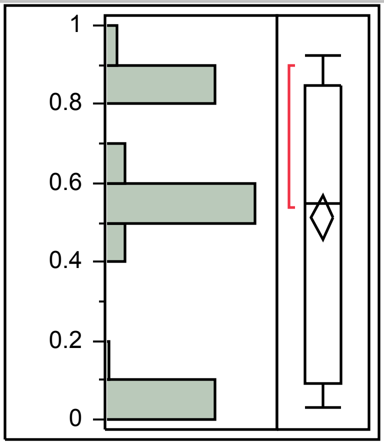


### Quantiles

| | | |
|---|---|---|
| 100.0% | maximum | 0.92319 |
| 99.5% | | 0.92319 |
| 97.5% | | 0.90975 |
| 90.0% | | 0.88744 |
| 75.0% | quartile | 0.8453 |
| 50.0% | median | 0.54835 |
| 25.0% | quartile | 0.09236 |
| 10.0% | | 0.0592 |
| 2.5% | | 0.04447 |
| 0.5% | | 0.03354 |
| 0.0% | minimum | 0.03354 |

### Summary Statistics

| | |
|---|---|
| Mean | 0.512484 |
| Std Dev | 0.3060069 |
| Std Err Mean | 0.0290449 |
| Upper 95% Mean | 0.5700441 |
| Lower 95% Mean | 0.4549238 |
| N | 111 |

## cg18239511

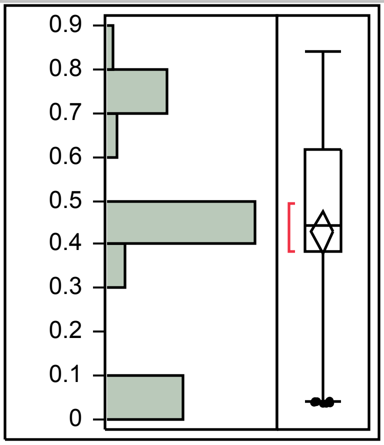


### Quantiles

| | | |
|---|---|---|
| 100.0% | maximum | 0.84075 |
| 99.5% | | 0.84075 |
| 97.5% | | 0.81715 |
| 90.0% | | 0.79096 |
| 75.0% | quartile | 0.61422 |
| 50.0% | median | 0.44372 |
| 25.0% | quartile | 0.38612 |
| 10.0% | | 0.04375 |
| 2.5% | | 0.03742 |
| 0.5% | | 0.0371 |
| 0.0% | minimum | 0.0371 |

### Summary Statistics

| | |
|---|---|
| Mean | 0.4283333 |
| Std Dev | 0.2533648 |
| Std Err Mean | 0.0240483 |
| Upper 95% Mean | 0.4759915 |
| Lower 95% Mean | 0.3806752 |
| N | 111 |

## cg13379757

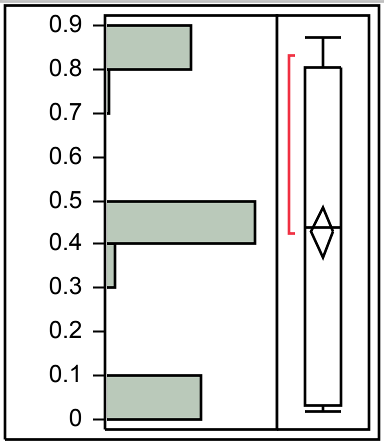


### Quantiles

| | | |
|---|---|---|
| 100.0% | maximum | 0.86942 |
| 99.5% | | 0.86942 |
| 97.5% | | 0.86213 |
| 90.0% | | 0.83793 |
| 75.0% | quartile | 0.80255 |
| 50.0% | median | 0.43787 |
| 25.0% | quartile | 0.03272 |
| 10.0% | | 0.0248 |
| 2.5% | | 0.02162 |
| 0.5% | | 0.02097 |
| 0.0% | minimum | 0.02097 |

### Summary Statistics

| | |
|---|---|
| Mean | 0.4273289 |
| Std Dev | 0.2985713 |
| Std Err Mean | 0.0283391 |
| Upper 95% Mean | 0.4834904 |
| Lower 95% Mean | 0.3711674 |
| N | 111 |

## Distributions

### cg19214707

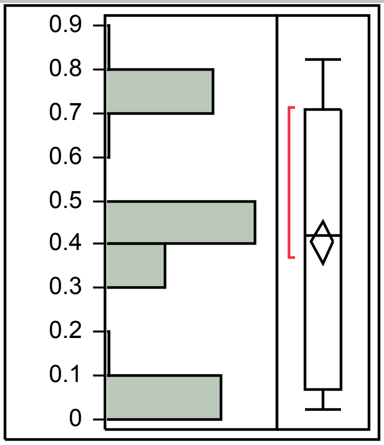


#### Quantiles

| | | |
|---|---|---|
| 100.0% | maximum | 0.81996 |
| 99.5% | | 0.81996 |
| 97.5% | | 0.78443 |
| 90.0% | | 0.76443 |
| 75.0% | quartile | 0.70594 |
| 50.0% | median | 0.42119 |
| 25.0% | quartile | 0.07034 |
| 10.0% | | 0.04137 |
| 2.5% | | 0.03034 |
| 0.5% | | 0.02338 |
| 0.0% | minimum | 0.02338 |

#### Summary Statistics

| | |
|---|---|
| Mean | 0.4048955 |
| Std Dev | 0.25875 |
| Std Err Mean | 0.0245595 |
| Upper 95% Mean | 0.4535666 |
| Lower 95% Mean | 0.3562244 |
| N | 111 |

### cg11019791

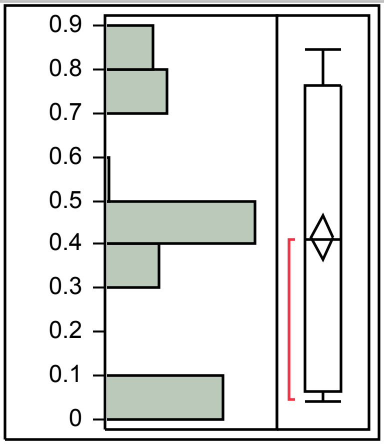


#### Quantiles

| | | |
|---|---|---|
| 100.0% | maximum | 0.84287 |
| 99.5% | | 0.84287 |
| 97.5% | | 0.83089 |
| 90.0% | | 0.81187 |
| 75.0% | quartile | 0.76394 |
| 50.0% | median | 0.41026 |
| 25.0% | quartile | 0.06542 |
| 10.0% | | 0.05234 |
| 2.5% | | 0.04581 |
| 0.5% | | 0.0411 |
| 0.0% | minimum | 0.0411 |

#### Summary Statistics

| | |
|---|---|
| Mean | 0.4154484 |
| Std Dev | 0.2725753 |
| Std Err Mean | 0.0258717 |
| Upper 95% Mean | 0.46672 |
| Lower 95% Mean | 0.3641767 |
| N | 111 |

### cg04506342

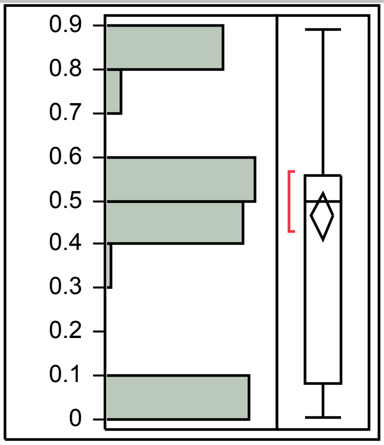


#### Quantiles

| | | |
|---|---|---|
| 100.0% | maximum | 0.89156 |
| 99.5% | | 0.89156 |
| 97.5% | | 0.86626 |
| 90.0% | | 0.83995 |
| 75.0% | quartile | 0.55777 |
| 50.0% | median | 0.4982 |
| 25.0% | quartile | 0.08272 |
| 10.0% | | 0.03809 |
| 2.5% | | 0.02092 |
| 0.5% | | 0.00585 |
| 0.0% | minimum | 0.00585 |

#### Summary Statistics

| | |
|---|---|
| Mean | 0.4639647 |
| Std Dev | 0.2792941 |
| Std Err Mean | 0.0265094 |
| Upper 95% Mean | 0.5165001 |
| Lower 95% Mean | 0.4114292 |
| N | 111 |

## Distributions

### cg25046571

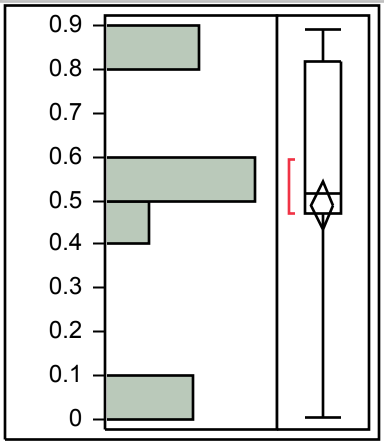


#### Quantiles

| | | |
|---|---|---|
| 100.0% | maximum | 0.88971 |
| 99.5% | | 0.88971 |
| 97.5% | | 0.88421 |
| 90.0% | | 0.86938 |
| 75.0% | quartile | 0.8184 |
| 50.0% | median | 0.51811 |
| 25.0% | quartile | 0.47242 |
| 10.0% | | 0.02604 |
| 2.5% | | 0.00908 |
| 0.5% | | 0.00694 |
| 0.0% | minimum | 0.00694 |

#### Summary Statistics

| | |
|---|---|
| Mean | 0.4902768 |
| Std Dev | 0.2934478 |
| Std Err Mean | 0.0278528 |
| Upper 95% Mean | 0.5454746 |
| Lower 95% Mean | 0.4350791 |
| N | 111 |

### cg10117599

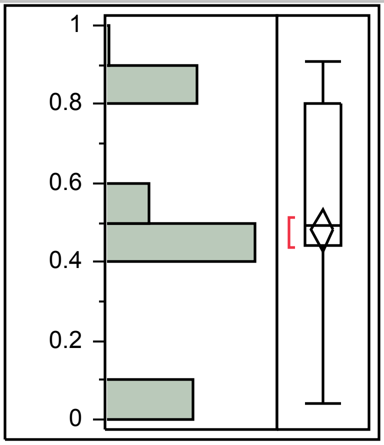


#### Quantiles

| | | |
|---|---|---|
| 100.0% | maximum | 0.90656 |
| 99.5% | | 0.90656 |
| 97.5% | | 0.893 |
| 90.0% | | 0.86868 |
| 75.0% | quartile | 0.80343 |
| 50.0% | median | 0.49096 |
| 25.0% | quartile | 0.44101 |
| 10.0% | | 0.05111 |
| 2.5% | | 0.04379 |
| 0.5% | | 0.03997 |
| 0.0% | minimum | 0.03997 |

#### Summary Statistics

| | |
|---|---|
| Mean | 0.4800515 |
| Std Dev | 0.2825167 |
| Std Err Mean | 0.0268153 |
| Upper 95% Mean | 0.5331932 |
| Lower 95% Mean | 0.4269099 |
| N | 111 |

### cg16398051

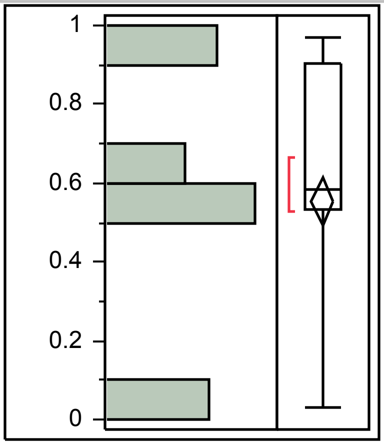


#### Quantiles

| | | |
|---|---|---|
| 100.0% | maximum | 0.9667 |
| 99.5% | | 0.9667 |
| 97.5% | | 0.95327 |
| 90.0% | | 0.94339 |
| 75.0% | quartile | 0.90495 |
| 50.0% | median | 0.58533 |
| 25.0% | quartile | 0.53292 |
| 10.0% | | 0.05444 |
| 2.5% | | 0.04266 |
| 0.5% | | 0.03071 |
| 0.0% | minimum | 0.03071 |

#### Summary Statistics

| | |
|---|---|
| Mean | 0.5536076 |
| Std Dev | 0.312121 |
| Std Err Mean | 0.0296252 |
| Upper 95% Mean | 0.6123178 |
| Lower 95% Mean | 0.4948973 |
| N | 111 |

## Distributions

### cg18514595

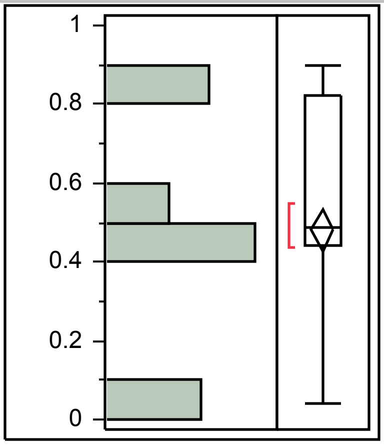


#### Quantiles

| | | |
|---|---|---|
| 100.0% | maximum | 0.89898 |
| 99.5% | | 0.89898 |
| 97.5% | | 0.88455 |
| 90.0% | | 0.87694 |
| 75.0% | quartile | 0.8221 |
| 50.0% | median | 0.48748 |
| 25.0% | quartile | 0.43925 |
| 10.0% | | 0.05092 |
| 2.5% | | 0.04256 |
| 0.5% | | 0.04093 |
| 0.0% | minimum | 0.04093 |

#### Summary Statistics

| | |
|---|---|
| Mean | 0.4801509 |
| Std Dev | 0.2858823 |
| Std Err Mean | 0.0271347 |
| Upper 95% Mean | 0.5339256 |
| Lower 95% Mean | 0.4263762 |
| N | 111 |

### cg16675926

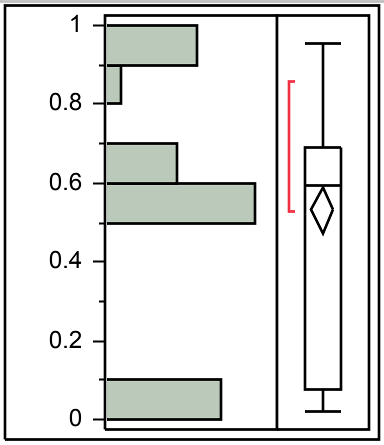


#### Quantiles

| | | |
|---|---|---|
| 100.0% | maximum | 0.9557 |
| 99.5% | | 0.9557 |
| 97.5% | | 0.95052 |
| 90.0% | | 0.93028 |
| 75.0% | quartile | 0.69087 |
| 50.0% | median | 0.59165 |
| 25.0% | quartile | 0.07822 |
| 10.0% | | 0.04588 |
| 2.5% | | 0.02898 |
| 0.5% | | 0.02176 |
| 0.0% | minimum | 0.02176 |

#### Summary Statistics

| | |
|---|---|
| Mean | 0.5305838 |
| Std Dev | 0.3186407 |
| Std Err Mean | 0.030244 |
| Upper 95% Mean | 0.5905204 |
| Lower 95% Mean | 0.4706472 |
| N | 111 |

### cg02299007

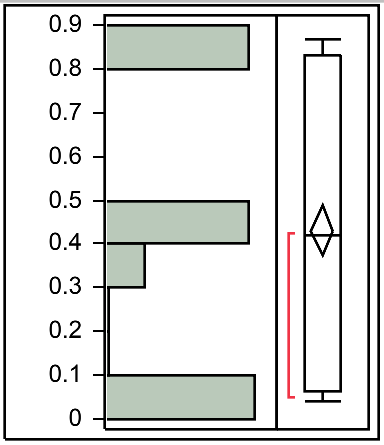


#### Quantiles

| | | |
|---|---|---|
| 100.0% | maximum | 0.86728 |
| 99.5% | | 0.86728 |
| 97.5% | | 0.86304 |
| 90.0% | | 0.8515 |
| 75.0% | quartile | 0.82933 |
| 50.0% | median | 0.42076 |
| 25.0% | quartile | 0.06368 |
| 10.0% | | 0.05574 |
| 2.5% | | 0.04842 |
| 0.5% | | 0.04305 |
| 0.0% | minimum | 0.04305 |

#### Summary Statistics

| | |
|---|---|
| Mean | 0.4313723 |
| Std Dev | 0.308615 |
| Std Err Mean | 0.0292924 |
| Upper 95% Mean | 0.4894231 |
| Lower 95% Mean | 0.3733216 |
| N | 111 |
